# Shared vision promotes family firm performance

**DOI:** 10.3389/fpsyg.2015.00646

**Published:** 2015-05-19

**Authors:** John E. Neff

**Affiliations:** Weatherhead School of Management, Case Western Reserve UniversityCleveland, OH, USA

**Keywords:** shared vision, family business, effective culture, firm performance, predictive model, family functionality, role clarity

## Abstract

A clear picture of the influential drivers of private family firm performance has proven to be an elusive target. The unique characteristics of private family owned firms necessitate a broader, non-financial approach to reveal firm performance drivers. This research study sought to specify and evaluate the themes that distinguish successful family firms from less successful family firms. In addition, this study explored the possibility that these themes collectively form an effective organizational culture that improves longer-term firm performance. At an organizational level of analysis, research findings identified four significant variables: Shared Vision (PNS), Role Clarity (RCL), Confidence in Management (CON), and Professional Networking (OLN) that positively impacted family firm financial performance. Shared Vision exhibited the strongest positive influence among the significant factors. In addition, Family Functionality (APGAR), the functional integrity of the family itself, exhibited a significant supporting role. Taken together, the variables collectively represent an effective family business culture (EFBC) that positively impacted the long-term financial sustainability of family owned firms. The index of effective family business culture also exhibited potential as a predictive non-financial model of family firm performance.

## Introduction

Family owned firms represent a significant portion of the U.S. economy, contributing nearly two-thirds of the gross domestic product, and employing 60% of the domestic workforce (Astrachan and Shanker, 2003); yet the field of family business research is relatively new (Bird et al., 2002). Most researchers take a particularly narrow, conventional approach to measuring the performance and predicting the success of family firms—often relying solely on financial data (Westhead and Cowling, 1998), which is rarely available for private family owned companies and often does not tell the whole story. While some analysts have attempted to use non-financial measurements to assess family firms, no one has created a multidimensional, non-financial assessment that measures the performance and predicts the sustainability of family owned companies.

This research attempts to break new ground by uncovering and understanding the precise portfolio of non-financial indicators that predict the long-term success of a family owned business. It is acknowledged that profit is necessary for the long-term sustainability of any business organization; however, research that uses profit maximization or ROI as the only measure of success of a family owned business usually falls short.

Prior research that relies on purely financial metrics is often limited to publicly traded companies, because of easily accessible financial performance data (Anderson and Reeb, 2003; Villalonga and Amit, 2006; Miller et al., 2007). Such research leaves out a huge population of companies, as most family owned businesses are privately held and rarely disclose financial details. Even when they do, the numbers may be misleading because many owners put non-financial benefits above profit (Tagiuri and Davis, 1992; Dunn, 1995; Paige and Littrell, 2002; Walker and Brown, 2004). Such measures also underestimate the role that culture plays in how a family business functions (Schein, 1996). Some multidimensional performance metrics, such as the balanced scorecard (Kaplan and Norton, 1992), paint a more complete, realistic picture of family firm performance, but tend to focus only on past performance.

Given the desire most family business owners have to keep their businesses alive for several generations (James, 1999), a predictive tool that measures current performance and the long-term sustainability of the family firm based on factors other than financial performance could be very useful to owners, as well as to researchers and advisors working with family owned companies (Neely et al., 1995; Corbetta and Salvato, 2004). Therefore, this research sets out to create such a tool by quantifying the findings of a previous qualitative study that identified several non-financial traits that, when combined, seemed to be associated with higher levels of organizational success (Neff, 2009). The present study uses a research technique similar to that used in previous research (Denison and Mishra, 1995) to explore whether specific cultural traits within an organization may be useful predictors of performance and effectiveness.

The findings from this study suggest that the performance of private family firms is, indeed, driven by a more complex set of priorities than those that drive their publicly traded and non-family owned counterparts. While financial success is certainly important to them, family firms appear to be highly motivated by non-financial goals—goals that reflect the complexity and interaction of the family and business systems (Davis and Tagiuri, 1989; Tagiuri and Davis, 1992). More recent research suggests that socio-emotional wealth may encompass the broad goals of family firms, rather than specific financial results such as firm profit maximization. In the family business context, socio-emotional wealth has been defined as the non-financial aspects of the firm that meet the family's affective needs, such as a sense of identity, perpetuation of the family firm, etc. (Gómez-Mejía et al., 2007).

### Nature and performance of family owned firms

The most distinguishing characteristic of the family owned business is the presence and interaction of the family system with the business system (Beckhard and Dyer, 1983; Kepner, 1983; Chua et al., 1999). The family's culture and the owners' non-financial motivations for being in business can have a profound effect on company performance—sometimes positive, sometimes negative (Dyer, 2006). For family firms to be sustainable, the relationship between the family and the business must be symbiotic and synergistic (Chua et al., 2003).

Unfortunately, there are very few details on the performance of private family owned companies because researchers tend to gravitate toward public family run companies, which are required to release financial data. Researchers also say that these firms tend to perform better than non-family owned firms in the United States (McConaughy et al., 1998; Anderson and Reeb, 2003; Lee, 2004, 2006; Villalonga and Amit, 2006). The opposite appears to be true in Europe and Asia (Claessens et al., 2002; Maury, 2006), perhaps because the definition of “family owned firm” varies from continent to continent (Westhead and Cowling, 1998; Miller et al., 2007). Further research by Miller et al. (2007) concluded that publicly traded family run businesses often get weaker once the founding member/generation is no longer in control (Miller et al., 2007).

Research on private family companies is less common, and the studies available are not always consistent with each other. What may be gleaned is that there is a wide spectrum of motivation among owners and managers and that the family personality (culture) can be a competitive advantage or disadvantage, depending on the circumstances. Emerging research also suggests that to evaluate the success of family owned companies by looking only at financial performance can distort the true value the business provides to the family. “Financial measures of family firms might be understated since they do not reflect the private benefits owners earn from their firms” (Astrachan and Zellweger, 2008, p. 7).

### Spectrum of motivation

There are families who are in business primarily to make a profit; however, other families may run their business mostly for the private benefit of the family or other non-economic outcomes. “Scholars have suggested that family firms display a strong preference toward non-economic outcomes such as autonomy, firm survival, or family harmony” (Astrachan and Zellweger, 2008, p. 11). One researcher has developed a tool to measure outcomes such as “family independence and satisfaction, tight-knit family, respect in the community and child and business development” (Mitchell et al., 2003). In a study of successful family owned companies, Tagiuri and Davis (1992) found that owners also put goals such as development of quality products, social advancement, good corporate citizenship, work-life balance, and job security on par with or ahead of profit goals.

The problem with these non-monetary goals, according to some researchers, is that they can “run counter to the optimal decisions for the business” (Bertrand and Schoar, 2006), dampening the bottom line, skewing the company's reported financial results, and shortchanging some minority and non-controlling shareholders. For example, family executives' emotional attachment to historical but unproductive assets or practices may negatively impact firm competitive advantage through delayed resource shedding decisions (Sharma and Manikutty, 2005). Still, others say that so-called “private benefits” are not necessarily detrimental to the firm itself or to minority shareholders, as some family owners are able to balance non-financial benefits and financial considerations effectively (Ehrhardt and Nowak, 2003).

Again, beyond these broad findings, there is little mention of or consensus on variables that will predict the success or failure of a family owned company. For example, in 2009, Yu et al. reviewed 212 research articles about family businesses studied over a 10-year period and found 259 different dependent variables (DVs) within seven interest domains, which included performance, strategy, environment, governance, succession, family roles, and family outcomes. So while research into family firms is becoming more prevalent, there remains a need for a holistic model that can appropriately analyze family firm performance (Sharma, 2004).

### Family firm culture

In addition to the owners' motivations, a family's culture can have a significant effect on how its business operates. In their book *In Search of Excellence*, Peters and Waterman (1982) popularized the notion that organizations have personality characteristics that can be harnessed as a competitive advantage (Lief and Denison, 2005). Previous research has indicated that organizational culture is particularly positive if it is valuable, rare, and difficult for other firms to duplicate (Barney, 1986; Zahra et al., 2004).

A company's culture typically starts with the founder and his/her vision and values, which can create a strong sense of shared purpose, identity, and destiny—keys to success in any business, family owned or not. It appears, though, that family owned companies tend to adhere to the founder's original purpose and that purpose can linger into future generations even after the founder's death (Denison et al., 2004). Such a strong cultural foundation can have a positive effect on the performance of a family owned business, but also it needs to be flexible (Denison et al., 2004). Next-generation owners and managers will bring their own talents and perspectives to the leadership role, and the culture that can adapt to the new style will be more likely to thrive (Eddleston, 2008).

### Long-term performance and family business sustainability

The sustainable family business model (SFBM), which guided this study, creates a framework for assessing the long-term performance of private family business. It is built on the paradigm of overlapping systems—family and business (Stafford et al., 1999). It also recognizes the unique dependency and interaction between the family system and the business system. The model suggests that the family owned business is a single system in which the complex dynamics inherent in how families operate will affect the performance and growth of the business (Olson et al., 2003). For example, the family may provide additional capital or labor to the business during times of financial distress. Also, the business may influence how the family members interact with each other. For example, the family may need to discuss what if any distributions are made from the business to family owners. According to the SFBM, for a family owned business to be sustainable, the business and the family must be successful (Stafford et al., 1999).

While the SFBM informed this study, the present research framework is more narrowly focused on the business system and the influences, organizational and familial, impacting long-term performance. The SFBM does not identify specific elements of the business system influencing long-term firm performance and sustainability. A significant goal of this study was to evaluate specific cultural characteristics of the business system.

Because the family system is a key and unique aspect of family businesses, the relationships among family members may have an influence on the long-term success of the business, as acknowledged by the SFBM. Further development of the SFBM recognizes that change or “disruptions” are natural and that the boundary between family and business is where adjustments are made (Werbel and Danes, 2010); therefore, the functionality of the family system may influence this adjustment process and the ultimate sustainability of the family business. Family businesses are heavily influenced by their founder and his or her vision for the business can exist across subsequent generations (Kelly et al., 2000). Such a vision, shared across the organization may play a role in the long-term success of the firm. Also because of the central role of the founder and family, a higher sense of trust and confidence in firm management may positively influence long-term performance. Inherent in the nature of family business, where family members juggle multiple roles (owner, employee, family member, etc.) the clarity in one's work role and lack of conflicting priorities may aid the long-term performance of the firm. Personal development, external learning opportunities, and a general commitment to learning may also aid the long-term performance of a family firm by improving human capital, raising awareness of the firm's external environment, and building a culture of continual learning. The next section builds on these themes and specifies the measures used in this research in order to identify specific firm characteristics that drive performance, particularly for private family firms.

## Material and methods

### Current research

**Figure 1:**
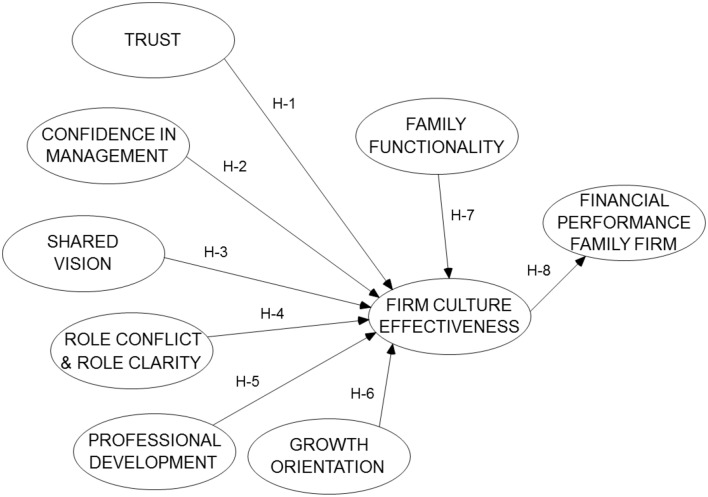
**Research model**.

The key questions driving this research are: What organizational traits influence financial performance in family owned firms? Do certain non-financial performance indicators align with perceived financial results of a family owned firm? Do these organizational traits compose a predictor variable of financial performance? The conceptual research model is shown in Figure 1. The model implies that both organizational traits and family traits influence an effective family business culture. The effective family business culture influences long term financial performance of the firm.

While the focus of this research is longer-term business sustainability, the model uses perceived firm performance as its dependent variable. The model accounts for the fact that profit is a necessary outcome, though not the only outcome, for a successful family owned business. In this model, non-financial organizational traits are components of family firm sustainability, and these factors align with and support sustained financial performance. Because the SFBM states that the business and the family must be strong, this study examines the organizational traits that may support the business system while including influence from the family system. The research model constructs are described below.

### Trust

Inherent in the nature of family owned businesses is the intense emotional connection among family members (Tagiuri and Davis, 1996) and when that connection is based on deep trust, the family system and the company benefit (Sundaramurthy, 2008). Trust is the foundation on which social capital is built (Bubolz, 2001). High trust within the family may reduce the transaction costs of exchange by lowering monitoring costs and opportunism (Steier, 2001).

Non-family employees believe the owning family is the firm (Neff, 2009); therefore, if the family is trustworthy, the business is trustworthy. This is different than conjectured by Sundaramurthy (2008, p. 95), who wrote, “… interpersonal trust cannot be sustained without confidence in the system that governs key interpersonal exchange.” The family may also serve as a “constellation of role models” within the firm (LaChapelle and Barnes, 1998). In this study, trust was measured with a scale adapted from Schoorman et al. (2007) as well as Mayer and Davis (1999). It included the sub-dimensions of benevolence and integrity.

*Hypothesis 1: Trust will have a positive influence on an Effective Family Business Culture*.

### Confidence in management

In a family business, employees' confidence in management is a key factor. It is distinct from the Trust hypothesis because it focuses on management's ability to achieve its stated objectives. Churchill and Hatten explain: “Trust in this sense involves knowing the goals or objectives another will try to attain. Confidence involves knowing the other is capable of attaining these objectives” (1997, p. 64).

While confidence in the ability of management is linked to trust, it may also reflect the level of experience and capability of family firm management. Management's capability to lead their organization may also be characterized as human capital that has a positive influence on family firm performance (Dyer, 2006). Also, if there is a positive sense of management's competence or ability, a higher sense of organizational efficacy may be present. Organizational efficacy, a firm level construct, may be considered equivalent to self-efficacy at the individual level and may be useful in examining organizational functioning at the strategic business level (Gist, 1987). In a study of suppliers to an American University in the Southwest, greater levels of organizational efficacy in family and non-family firms were associated with higher performance (Stanley and McDowell, 2014).

In the qualitative study that informed this research, confidence in the abilities of other employees was a distinct theme, separate from the trust between family and non-family employees; therefore, the Confidence in Management construct was separated from other elements relating to trust. It focuses on the assessment of the ability of the firm's leadership or top management team to be successful. Confidence in management ability was measured by a scale adapted from Mayer and Davis (1999).

*Hypothesis 2: Confidence in Management will have a positive influence on an Effective Family Business Culture*.

### Shared vision

Shared Vision is central to the long-term success of any organization. The idea of an organizational Shared Vision was first articulated by Senge as an important aspect of maintaining a vibrant and successful organization over the long run (Senge, 1990). Shared vision bonds organizational members together through a common desired future. Value-laden visions were associated with greater affective organizational commitment among organizational members (Dvir et al., 2004). The aspirational nature of such a Shared Vision also directs the energy of the organization in a positive manner. A Shared Vision inspires the entire organization to hopefulness and success (Boyatzis and McKee, 2005).

Managing through a Shared Vision can have a wide-ranging positive impact on an organization—improving performance, promoting change, providing a foundation for a strategic plan, motivating individuals, and providing a context for decisions (Lipton, 1996). Other research suggests that Shared Vision occupies a core role in the team innovation process (Pearce and Ensley, 2004), plays a role in promoting extra-role or championing behavior in mergers and acquisitions (Clayton, 2009), amplifies the impact of emotional intelligence in both IT team engagement (Mahon, 2008), and physician leadership (Quinn, 2012).

Shared Vision is also critical in the family business context. According to Ward (1997), “… the best practice that is most important to long-term family business growth is [defining] family purpose and mission, family values, and the motivations and rationale for continued business ownership” (p. 335).

According to Lansberg (1988) and Ward (1997), owners should explicitly communicate their succession plan, painting a clear, viable picture of what the company will look like once the next generation takes control (Lansberg and Astrachan, 1994), especially if more than one member will be in a leadership position (Hoy and Verser, 1994). Including members of that next generation in the strategic planning process is also critical to keeping the family's vision alive (Mazzola et al., 2008). Shared Vision can also be the guiding force in the strategic renewal of family firms (Boyatzis and Soler, 2012). Shared Vision not only strengthens the company, it can unite family members—whether or not they are employed in the business—and can reduce unproductive conflict among family in the firm (Kellermanns and Eddleston, 2004).

The scale used to evaluate the Shared Vision construct is from Boyatzis (2008). The complete scale consists of three dimensions: Shared Vision, Compassion, and Overall Positive Mood (Boyatzis, 2008). This research project used only the Shared Vision portion of the scale.

*Hypothesis 3: Shared Vision will have a positive influence on an Effective Family Business Culture*.

### Role clarity/role conflict

Role clarity results from clear behavioral and performance expectations for a work role and role conflict results from incompatibility of a work role with personal values or multiple roles that conflict with each other (van Sell et al., 1981). Role clarity and role conflict have been extensively studied and research has linked them to a variety of correlates including job performance (Tubre and Collins, 2000). In the family business context, it is common for family member employees to be confused about their roles in the company. Members often play several roles simultaneously—such as owner, employee, manager, parent, sibling, child, etc. (Gersick et al., 1997). In this complex environment, the expectations of these roles may not be clear, or may even be in conflict (Sundaramurthy and Kreiner, 2008). Indeed, family businesses face conflict from many sources, including role ambiguity and role conflict (Harvey and Evans, 1994). For some roles, family harmony may be more important, while for others, return on investment may take precedence. Such role conflict (Memili et al., 2013) and/or lack of clarity may interfere with family business performance.

The Role Clarity/Role Conflict scale used in this research was originally developed by Rizzo and House (Rizzo et al., 1970). It should be noted that in their original conceptualization, Rizzo and House refer to role conflict and role ambiguity. For this study, role ambiguity has been re-characterized using the term role clarity. The original role ambiguity scale items, all refer to clarity rather than ambiguity; therefore, the construct label has been changed to Role Clarity to align with the scale items and avoid reverse scoring. While this characterization is not common, the inconsistency between the construct label and item wording has been noted in previous research (Tracy and Johnson, 1981). The scales are designed to capture the extent to which individuals may understand what is expected of them (clarity) and whether or not the roles they play are inconsistent with their own values or with each other (conflict) (van Sell et al., 1981).

*Hypothesis 4: Role Clarity/Role Conflict will have a positive/negative influence, respectively, on an Effective Family Business Culture*.

### Professional development and networking

The education and experience that individuals bring to an organization can affect how successful the organization is—so can the extent to which they are able to continue to learn and grow. This research focused on two sub-dimensions of organizational development: perception of the firm's commitment to human capital development and the extent to which employees engage in personal development through professional networking to develop relationships with industry peers and other members of the community. On an individual level, engagement in professional activities is associated with career success (Forret and Dougherty, 2004). Family owner/managers who are connected to people and resources outside the family—including customers, suppliers, and other industry participants—may be able to stay abreast of valuable market intelligence and new business opportunities. Resources for the ultimate success of the family firm are constrained by relying too heavily on the limited human capital stock of the family (Sirmon and Hitt, 2003). The scales utilized to capture organizational development were adapted from the commitment to organizational learning scale (Calantone et al., 2002) and the networking behavior scale (Forret and Dougherty, 2001).

*Hypothesis 5: Professional Development and Networking will have a positive influence on an Effective Family Business Culture*.

### Growth orientation

A firm's orientation toward growth may be a factor in its long run performance. For example, the growth aspirations of small business managers were associated with actual growth (Wiklund and Shepherd, 2003), and this effect was enhanced by higher manager education and experience. In the family firm context, some long lived family firms survived without growth but their survival was attributed to special circumstances; including tightly controlled ownership, stable competitive environments, and little technological change (Ward, 1997). With little a priori knowledge of these conditions, growth may be necessary to avoid stagnation and decline of a family business. While family firms face special challenges to achieve growth (Ward, 1997), Miller et al. (2008) found no difference in market growth expectations and actual growth between small private family firms and their non-family counterparts (Miller et al., 2008). This construct seeks to measure perceptions of the organization's capacity to grow and management's ability to spur that growth. This construct also measures perceptions of certain performance indicators: sales volume, employment growth, and investment in capacity/technology.

The Growth Orientation scale used in this research comes from Poza et al. (2004). It is anticipated that Growth Orientation within family firms would be positively associated with financial performance and would influence the overall effectiveness of the organization. In addition, other included items related to the perceived importance of common performance metrics, such as sales volume, employment, and investment for the future, as adapted from previous research (Rutherford et al., 2006).

*Hypothesis 6: Growth Orientation will have a positive influence on an Effective Family Business Culture*.

### Family functionality

Fundamental to the SFBM is the recognition of the interplay between family and business. To capture the influence of the family on the business, the family APGAR scale is used to measure family functionality. This scale was originally developed in a clinical setting to assess the functional integrity of families and the functional health of patients' families. The APGAR acronym comes from the five functional components of adaptability, partnership, growth, affection, and resolve. The instrument measures a person's satisfaction with the five basic components of family function (Smilkstein, 1978). The APGAR instrument revealed a Cronbach-alpha (CA) of 0.82 in its original research assessment.

While the APGAR scale was developed in the medical field, it has been used in family business research (Danes et al., 1999; Avery et al., 2000; Danes and Olson, 2003; Danes and Lee, 2004). While it is not a complete measure of family success as described in the SFBM, a certain level of family functionality is necessary to avoid any negative effect of the family on the business. Also, higher levels of family functionality as measured by the family APGAR were associated with greater success in achieving the family's most important family goal (Danes et al., 1999).

*Hypothesis 7: Family Functionality will have a positive influence an Effective Family Business Culture*.

### Firm culture effectiveness

This is a second-order construct of the seven independent constructs discussed above. The hypothesis suggests that the independent variables of Trust, Confidence in Management, Shared Vision, Role Clarity/Role Conflict, Professional Development, Growth Orientation, and Family Functionality collectively form a composite second-order construct. This research seeks to test whether this construct, which is termed “Effective Family Business Culture” (EFBC), will have a positive effect on the financial performance of family owned firms.

A key aspect of this research is to investigate the EFBC construct as a composite indicator of firm financial performance in the context of family owned firms. It may be that the EFBC is a second-order formative construct. In that case, it is anticipated that the model independent variable constructs cause EFBC rather than reflect its presence. The independent variable constructs such as Growth Orientation, Shared Vision, and Family Functionality may be seen as influencing EFBC. It may not be as clear if other constructs, such as Trust or Confidence in Management, cause or reflect EFBC. Beyond the direction of causality, other factors are indicative of a formative relationship. The components of formative constructs may not necessarily co-vary as with reflective indicators and will be examined during survey data analysis. Also, formative indicators are not interchangeable, and the removal of one or more indicators can alter the nature of the formative construct. In addition, the antecedents of formative indicators may not align as they should with reflective indicators (Jarvis et al., 2003).

*Hypothesis 8: An Effective Family Business Culture will have a positive influence on the Financial Performance of a Family Business*.

Table 1 summarizes the testable research hypotheses included in the model.

**Table 1:** **Research hypotheses**.

**Hypotheses**
H1: Trust will have a positive influence on an Effective Family Business Culture.
H2: Confidence in Management will have a positive influence on an Effective Family Business Culture.
H3: Shared Vision will have a positive influence on an Effective Family Business Culture.
H4: Role Clarity/Role Conflict will have a positive/negative influence, respectively, on an Effective Family Business Culture.
H5: Professional Development and Networking will have a positive influence on an Effective Family Business Culture.
H6: Growth Orientation will have a positive influence on an Effective Family Business Culture.
H7: Family Functionality will have a positive influence on an Effective Family Business Culture.
H8: Effective Family Business Culture will have a positive influence on the Financial Performance of a Family Business.

### Control variables

It has been widely discussed that family firms vary in size and complexity. To address potential influence, this study included both firm size, as measured by total employment, and firm age, as indicated by each firm's founding year, as control variables. In addition, firm financial performance may also be influenced by its particular industry; therefore, industry is included as a control variable using the North American Industry Classification System (NAICS).

### Data collection and preparation

Research participants were identified from private databases, and responses were collected through an online survey. The three main national databases included a graphic arts industry publication, a commercial business database service, and a list of private firms solicited by an online survey company. This approach may lessen selection bias issues when using a convenience sample. The ratio of family owned and operated businesses to non-family owned businesses in any of the databases was not known. Other researchers have estimated that the overall percentage of family owned businesses (public and private partnerships and corporations) in the United States is approximately 60% (Astrachan and Shanker, 2003).

### Survey response rates

The graphic arts industry database maintains an opt-in e-mail list of approximately 7200 individuals. While the ratio of family owned and managed firms to non-family owned firms was not available, a recently published figure from the Printing Industry Association indicated that, on average, 60% of printing firms characterize themselves as family-run enterprises. Based on this statistic, the e-mail list would have approximately 4320 family firms. Survey links were emailed in three phases, approximately 2 weeks apart. A total of 47 responses were received, 37 of which were complete, resulting in response rates of 1.1 and 0.9%, respectively.

To mine the commercial business database, software provided allowed the search of each firm's profile for such phrases as “family firm” or “family business.” This resulted in a list of 1229 companies that were sent a letter directing interested participants to an online survey. Later, two reminder postcards were sent at 10-day intervals. Sixty-four were returned as undeliverable; 33 firms responded that they were not family owned businesses; and eight declined to participate in the survey. A total of 43 companies completed the survey, and 15 partially completed it. Based on all responses received from these mailings, the proportion of family owned and managed firms on the list would be approximately 67%. The surveys returned suggest an overall response rate of 7.4%. Forty-three of the surveys received were usable, resulting in a final 5.5% response rate.

The third set of responses came from the online survey company, which asked 1300 owner-run private businesses to participate in this research and provided responses from 79 companies. Of the 79, 28 did not self-classify as family managed, and three only partially completed the survey. This left 48 completed surveys from companies that self-identified as family firms. Based on the survey response, the implied percentage of family firms in this sample would be 64.5%. If this percentage of family owned and managed firms applied to the entire group of 1300 solicited firms, then 838 firms would fit the criteria of this study; therefore, the 48 completed surveys would represent a response rate of 5.7%.

The final tally of completed surveys included 110 firms, with a single respondent per firm. It should be noted that the overall response rate in this study was low; however, this seems to be an issue for this field of study, and low participation rates for surveys of private family firms are common (Winter et al., 1998). Reasons cited for low response rates include a reluctance to divulge financial details, difficulty in identifying private family firms, and a difficulty identifying appropriate participants within such firms. The Winter et al. (1998) study cited a 1997 Arthur Anderson/MassMutual national survey of family businesses that reported a response rate of 10.3%. The Winter et al. (1998) study also reported that prior surveys by MassMutual in 1993, 1994, and 1995 had even lower response rates.

Data collection began in October 2009 and ended in April 2010. Two of the three data sources did not permit follow up with non-respondents. Non-respondents from the third data source gave such reasons as “no interest in participating,” “lack of time to dedicate to completing the survey,” and “a policy of non-disclosure of firm financial information.”

### Sample

The research sample consisted of 110 senior executives from firms that self-identified as being family owned and having family members active in firm management. Table 2 details both the respondent and firm characteristics.

**Table 2:** **Survey respondent and firm characteristics**.

**Level of analysis**	**Characteristic**	**Sample data**
Respondent	Gender	71.8% Male 28.2% Female
	Age	53.6% Older than 50 years old 20.9% Between 40 and 50 years old 25.5% Younger than 40 years old
Firm	Generation	32.7% First generation 32.7% Second generation 34.6% Third generation
	Ownership	80.4% Fewer than five owners 19.6% More than five owners
	Voting control (owns >50% voting)	57.9% Single person 42.1% More than one person
	Family employees	81.1% Fewer than five family employees 18.9% Five of more family employees
	Industry (NAICS codes)	30.3% Manufacturing 17.4% Retail 10.1% Wholesale trade 8.3% Construction 6.4% Fin/Ins/Real Estate 2.8% Transport/Warehouse 1.8% IT services 22.9% Other
	# of Employees	70.6% Fewer than 50 14.7% Between 50 and 250 14.7% More than 250

### Measures

The survey contained 110 questions and all of the independent variable construct items were adapted from previously established scales. See the Appendix in Supplementary Material for a complete list of survey items by construct, with references to their sources. All independent variables used a five-point Likert scale. The dependent variable items, also in the Appendix in Supplementary Material, related to multiple facets of performance, including sales growth, profit level, and overall firm growth over a 3-year period. The dependent variables used a seven-point Likert scale. Respondent perceptions of their firm's recent 3-year performance were contrasted against perceived long-term trends of their own firms as well as against perceived performance of major competitors. The study intended to conduct a broad assessment of firm performance across multiple dimensions and from various perspectives in order to achieve a more holistic measure of performance.

## Results

### Data collection and analysis

Study methodology included data screening and exploratory factor analysis (EFA), using SPSS, and structural equation modeling using partial least squares (PLS). PLS works well with smaller sample sizes and for the inclusion of formative constructs. PLS also is useful in analyzing data that do not conform to the restrictive statistical assumptions of other analysis techniques. Finally, PLS is useful in developing predictive models (Chin, 1998). A bootstrapping technique, with 500 resamples, tested the significance of path coefficients (Chin, 1998). Bootstrapping is a non-parametric technique built into PLS to improve model estimation by calculating sampling error and generating *t*-values (Lowry and Gaskin, 2014).

### Missing values and normalcy of the data

In almost all cases, missing values were replaced by the mean of the particular item. Several independent variables (nine of 74) had missing values in excess of 10% of the total responses; however, for one particular construct, the benevolence sub-dimension of the Trust scale, all six construct items had very high missing values, ranging from 27.3 to 39.1%. These particular questions dealt with an employee's relationship to his or her supervisor and may have been confusing to owner-operators who do not have a direct supervisor. These items were excluded from further analysis.

An analysis of the independent variable items indicates the presence of non-normal data. Forty of 74 items had standardized skewness values in excess of ± 3.00, indicating a fairly high degree of non-normality. Standardized values of Kurtosis were less so, with 13 of 74 items in excess of ± 3.00. Further tests using the Kolmogorov–Smirnov and Shapiro–Wilkes normality tests also suggest non-normal data, as all items for both tests were significant at the 0.001 level.

### Exploratory factor analysis (first-order factors)

Due to the relatively high number of constructs and survey items compared to the sample size of 110, an EFA on all constructs could not be performed simultaneously. As most of the independent variable constructs have been previously established, partial EFA was used to verify the validity of the constructs in the context of this research. Principle axis factoring (PAF) was employed for the EFA extraction method to evaluate the first-order independent variable constructs. Oblique rotation using Promax was used to account for potential correlation of items within a given construct (Field, 2005).

The initial EFA assessed the construct items for suitability in this research, as exhibited by high factor loadings (0.60 and above) and low cross loadings with other construct items (no cross loadings above 90% of factor loading). The results are in Table 3. More than 79% of the items (53 of 67) exhibited moderate to high factor loadings in excess of 0.40. Six of the nine cross-loaded items involved reverse-scored questions, which can cloud EFA results. Given that all scales are established constructs from prior research, it was decided not to alter the item makeup based on the EFA outcomes; however, two of the constructs, Growth Orientation (GO) and Signs of Growth (GOs), showed cross loadings among two of four and one of three items, respectively. These constructs may warrant close monitoring in subsequent analysis.

**Table 3:** **Summary EFA analysis on first-order constructs**.

**EFA group**	**Construct name (Abbreviation)**	**KMO**	**Bartlett sphericity**	**Variance explained (%)**	**Total # of items**	**# of Factor Wts. >0.60**	**# of Factors >0.40 and <0.60**	**# of Factors <0.40**	**Cross loading >0.90**
1	Role clarity (RCL)	0.825	0.000	52.2	6	4	1	1	0
1	Role conflict (RCN)				7	4	3	0	0
2	Confidence in Mgt. (CON)	0.924	0.000	64.4	6	6	0	0	0
2	Trust integrity (TI)				6	5	0	1	1^*^
3	Commit org. learning (OLC)	0.802	0.000	52.6	6	4	1	1	0
3	Professional networking (OLN)				4	2	1	1	0
4	PEA – compassion (PNC)	0.854	0.000	61.9	6	1	2	2	3^*^
4	PEA – overall positive mood (PNM)				6	4	0	2	2^*^
4	PEA – Shared Vision (PNS)				8	4	2	2	0
5	Family functionality (APGAR)	0.783	0.000	57.6%	5	3	2	0	0
5	Growth orientation (GO)				4	2	0	2	2
5	Signs of growth (GOs)				3	2	0	1	1

* *Involves reverse scored items*.

In addition, constructs were evaluated using Cronbach-alpha (CA), in Table 4. This analysis indicates that with one exception, CA-values for the research constructs exceeded 0.70, and most exceeded 0.80, which indicates a high level of convergent validity among the independent variable construct items. The lone exception involved the GOs construct, which had a CA-value of 0.438, well below a common threshold of 0.70 (Field, 2005). In addition, the elimination of the most problematic items in this scale only marginally improved the overall CA; therefore, the GOs construct was eliminated from subsequent analyses.

**Table 4:** **Construct reliability**.

**Construct name (Abbreviation)**	**Cronbach alpha**	**Improved by**	**If delete item…**
Role clarity (RCL)	0.819	n.a.	n.a.
Role conflict (RCN)	0.863	n.a.	n.a.
Confidence in management (CON)	0.907	n.a.	n.a.
Trust integrity (TI)	0.854	0.007	TI4r
Commit org. learning (OLC)	0.792	n.a.	n.a.
Professional networking (OLN)	0.725	n.a.	n.a.
PEA – compassion (PNC)	0.801	n.a.	n.a.
PEA – overall positive mood (PNM)	0.884	n.a.	n.a.
PEA – shared vision (PNS)	0.860	0.003	PNS2
Family functionality (APGAR)	0.812	0.007	APRG5
Growth orientation (GO)	0.759	n.a.	n.a.
Signs of growth (GOs)	0.438	0.050	GOs2

### Measurement model

**Figure 2:**
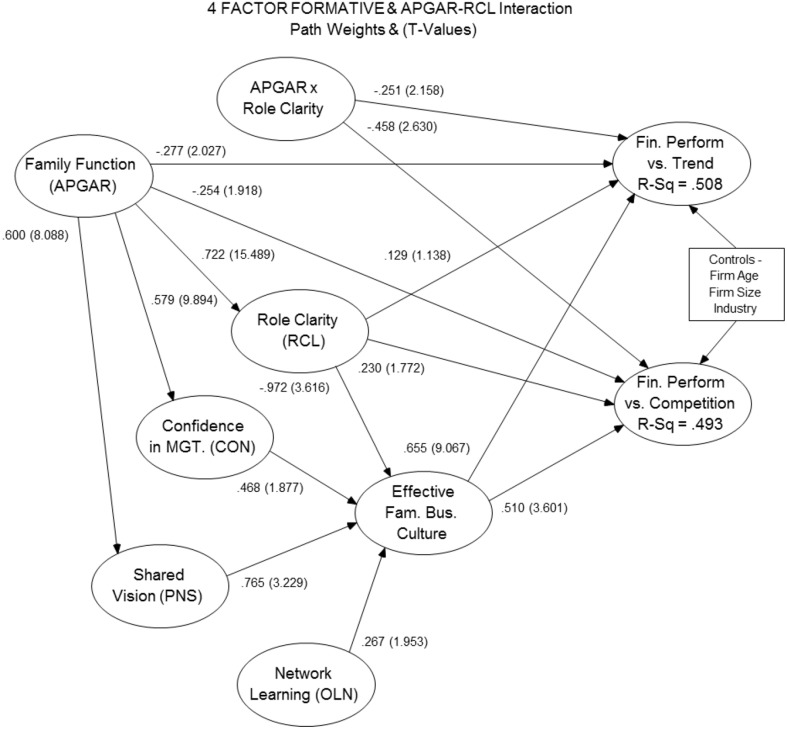
**Final structural model**.

This research sought to identify non-financial drivers of family firm financial performance. As a result, the focus was on the explanatory power of the first- and second-order constructs on the firm performance DVs. From the initial model in Figure 1, all non-significant paths were removed from the independent variables to the second-order construct, EFBC. In the final model, see Figure 2, five first-order independent variables remain along with the EFBC construct. The APGAR construct exhibited no direct significant effect on the EFBC construct; however, the APGAR construct had a highly significant and positive effect on the first-order independent variables; therefore, its influence on EFBC appears to be fully mediated through the Shared Vision (PNS), Role Clarity (RCL), and Confidence in Management (CON).

Based on the final model, the results from the tested hypotheses are summarized in Table 5.

**Table 5:** **Hypotheses test outcomes**.

**Hypothesis**	**Model outcome**	**Performance model hypotheses**
H1	Not supported	Trust will have a positive influence on an Effective Family Business Culture.
H2	Supported	Confidence in Management will have a positive influence on an Effective Family Business Culture.
H3	Supported	Shared Vision will have a positive influence on Effective Family Business Culture.
H4	Not supported	Role Clarity/Role Conflict will have a positive/negative influence, respectively, on an Effective Family Business Culture.
H5	Partially supported	Organizational Development and Professional Networking will have a positive influence on an Effective Family Business Culture.
H6	Not supported	Growth Orientation will have a positive influence on an Effective Family Business Culture.
H7	Not supported	Family Functionality will have a positive influence on an Effective Family Business Culture.
H8	Supported	Effective Family Business Culture will have a positive influence on the Financial Performance of a Family Business.

### Convergent and discriminate validity

As seen in Table 6, the independent variable constructs exhibited high composite reliability in excess of 0.80, which indicates an acceptable level of scale reliability. Table 7 reports the between-construct correlations along with the Average Variance Explained (AVE) square root (bold diagonal values). For the most part, the values indicate clear discriminate validity among the independent variable constructs; however, two variable pairs did not exhibit clear differences. These pairs included the Shared Vision to Confidence in Management, as well as Role Clarity to Confidence in Management variables. This is an area of concern when considering the possible impact on the predictive potential of the final model. Unclear discriminant validity suggests the possibility of redundant constructs. As noted in further analysis below, the final model did not exhibit multicolinearity among the independent variables.

**Table 6:** **Convergent and discriminant validity**.

**Construct (abbreviation)**	**Average variance explained (AVE)**	**Composite reliability**	**R-square**	**Cronbach alpha**	**Communality**	**Redundancy**
Family functionality (APGAR)	0.557	0.862	–	0.799	0.557	–
Confidence in management (CON)	0.667	0.923	0.335	0.899	0.667	0.222
Professional networking (OLN)	0.543	0.824	–	0.719	0.543	–
Shared vision (PNS)	0.502	0.887	0.361	0.851	0.502	0.179
Role clarity (RCL)	0.518	0.863	0.521	0.808	0.518	0.259
Effective family business culture (EFBC)	–	–	0.936	–	0.129	0.115
Perf. vs. comp.	–	–	0.493	–	0.242	(0.012)
Perf. vs. trend	–	–	0.508	–	0.345	(0.024)

**Table 7:** **Construct correlations matrix**.

**Construct**	**APGAR**	**CON**	**OLN**	**PNS**	**RCL**	**Eff. FB culture**	**Perf. vs. comp**.	**Perf. vs. trend**
APGAR	**0.747**							
CON	0.579	**0.817**						
OLN	0.172	0.289	**0.737**					
PNS	0.600	0.747	0.405	**0.708**				
RCL	0.722	0.703	0.154	0.630	**0.720**			
EFBC	0.082	0.433	0.562	0.610	−0.120	–		
Perf. vs. comp.	−0.022	0.252	.249	0.314	0.003	0.504	–	
Perf. vs. trend	−0.083	0.211	.336	0.330	−0.115	0.649	0.594	–

*Square root of AVE on bold diagonal. APGAR, family functionality; CON, confidence in management; OLN, professional networking; PNS, shared vision; RCL, role clarity; EFBC, effective family business culture; Perf. vs. comp., Firm performance vs. competition; Perf. vs. trend, firm performance vs. historical trend*.

### Interaction effects

The final model also indicates a significant interaction effect between Family Functionality (APGAR) and Role Clarity (RCL) variables. The paths from the interaction term to the dependent variables have negative path weights and are both significant at the 0.05 level, supporting the presence of a moderating effect. The strength of the moderating effect can be assessed by comparing the model R-Squared with and without the moderating variable (Henseler and Fassott, 2010). The moderating variable seems to have a nearly moderate level impact of 0.132 on the performance-trend dependent variable, and 0.158 on the performance-competition dependent variable, based on criteria described in previous research (Chin et al., 2003).

**Figure 3:**
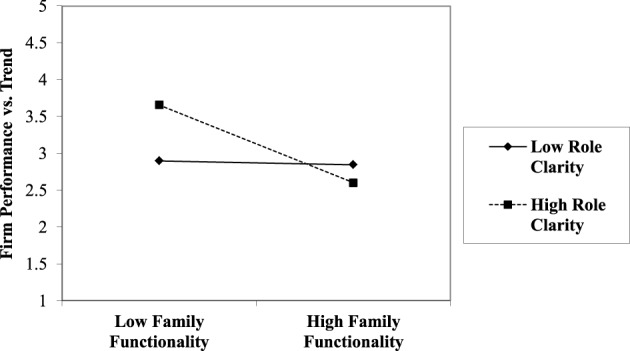
**The effect of perceived family functionality on perceived relative firm performance vs. historical trend by low and high role clarity**.

**Figure 4:**
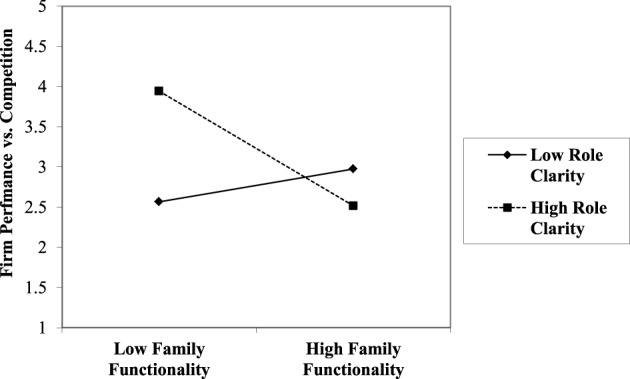
**The effect of perceived family functionality on perceived relative firm performance vs. competition by low and high role clarity**.

Using a two-way graphic, interpretation of this interaction is more clearly illustrated. In Figures 3, 4, high levels of Role Clarity (RCL) in the presence of low Family Functionality (APGAR) yield higher levels of firm financial performance. While in the simultaneous presence of high Role Clarity combined with high Family Functionality, firm performance is slightly reduced. The data also indicate that when Role Clarity is low, the addition of high Family Functionality has only a slight positive effect on firm performance.

### Model predictive value

One of the advantages of PLS is in developing predictive models. As with other regression-based approaches, the final model R-Squared magnitude of more than 50% is one indication of the explanatory potential of the research model. The PLS blindfolding procedure, based on the Stone–Geisser test, can further evaluate the predictive validity of the final research model. Table 8 indicates blindfold test results greater than zero, suggesting the final structural model has some predictive relevance (Chin, 2010).

**Table 8:** **Construct cross-validated redundancy**.

	**SSO**	**SSE**	**1-SEE/SSO**
CON	660.000	520.006	0.212
PNS	880.000	739.222	0.160
RCL	660.000	511.329	0.225
EFBC	2640.000	2257.967	0.145
Perf. vs. comp.	660.000	549.304	0.168
Perf. vs. trend	660.000	606.466	0.081

*CON, confidence in management; PNS, shared vision; RCL, role clarity; EFBC, Effective family business culture; Perf. vs. comp., firm performance vs. competition; Perf. vs. trend, firm performance vs. historical trend; SSO, sum of squares observed; SSE, sum of squares error*.

In PLS modeling with formative constructs, multicolinearity can be a significant issue. High levels of multicolinearity among the components of a formative index imply a redundancy among index variables and cloud assessment of the influence of a particular variable. Among the four independent variables that compose an EFBC, the second-order formative construct—the maximum variance inflation factor came to 2.884, as seen in Table 9, and is well below the common cut-off threshold value of 10 (Diamantopoulos and Winklhofer, 2001); therefore, multicolinearity did not affect the inclusion of the four independent variables. So despite the lack of clear discriminant validity among some of the independent variables, the lack of multicolinearity indicates little construct redundancy in the final model.

**Table 9:** **Variance inflation factors**.

**Dependent variables**	**Independent variables**			
	**CON**	**OLN**	**PNS**	**RCL**
CON	–	2.003	1.204	1.739
OLN	2.884	–	2.366	2.210
PNS	2.202	1.086	–	2.093
RCL	2.238	2.450	1.187	–

*CON, confidence in management; OLN, professional networking; PNS, shared vision; RCL, role clarity*.

## Discussion

The objective of this study was to investigate potential organizational drivers of financial performance in private family owned and managed companies through multivariate statistical techniques as suggested by Westhead and Cowling (1998). This research contributes to the understanding of family firm performance by using PLS and complex constructs operationalized at a higher level of abstraction (Sarstedt et al., 2014). The initial research model hypothesized that multiple independent variable constructs form a second-order formative factor, which can influence overall firm financial performance. The first of three findings of this research project involves the significant influence of a new second-order formative construct, effective family business culture (EFBC). Similar to Denison and Mishra (1995), the EFBC construct is focused on cultural traits that influence long term family firm performance and is not fully inclusive of the domain of family firm culture (Denison and Mishra, 1995). Analysis indicated that EFBC is highly significant and has a strong positive effect on overall firm performance. As a formative construct, the non-financial EFBC could be useful in predicting family firm performance since objective financial data is difficult to obtain from private family firms (Mazzi, 2011; Carney et al., 2013). Relatively good results from the variance inflation factor and blindfold tests support the potential predictive power of this construct.

Another research contribution is the identification of the specific components of EFBC. These included four first-order reflective constructs: Confidence in Management (CON), Shared Vision (PNS), Role Clarity (RCL), and Professional Networking (OLN). The constructs did not exhibit excessive cross correlation to the EFBC construct and were significant at the 0.10 level or higher. Shared Vision exhibited strong significance as well as the highest positive impact on EFBC. Two of the remaining three independent variables (CON and OLN) were found to have a positive influence on EFBC; however, the role clarity construct (RCL) revealed a negative influence on EFBC, contrary to initial expectations. This surprising finding suggests that Role Clarity in the context of family firms has a dampening effect on effective culture and, thus, firm performance. In addition, the integrity dimension of trust did not exhibit a significant impact on EFBC and therefore did not support the first hypothesis. While some research has linked integrity as a component of trust, with firm performance (Davis et al., 2000), the unique context of family business may require a more nuanced approach. Trust in family firms may be different than non-family firms due to the presence of familial relationship-based trust (Sundaramurthy, 2008). Schoorman et al. (2007) suggest that it may be appropriate to specify additional model elements in unique contextual settings. Family business may represent such a setting and further investigation may be needed to better understand the relationship between the elements of trust and firm performance.

The conceptual interpretation of the formative construct, EFBC, shows an interesting variety of components. Shared Vision, representing a desired future, shared across the organization, may represent a foundational element, giving direction and energy toward a desired and common organizational future. Other research found that Shared Vision influences firm performance (Calantone et al., 2002). Confidence in Management ability, suggests the importance of having talent and experience to achieve that vision within the organization's management team. Owners with management and industry experience are positively associated with firm performance (Dyke et al., 1992). In their study, Dyke et al. (1992) also found that having business owner parents was not associated with higher firm performance; though the authors did not address family business successor owners. Family firms with the intention of keeping the business in the family have greater opportunity to develop successor owner/mangers, for example, by involving the next generation in strategic planning (Mazzola et al., 2008). Role Clarity is typically viewed as a positive attribute and on an individual level is positively related to job performance (Tubre and Collins, 2000). Other research indicates that a lack of Role Clarity is a source of tension among business-owning couples (Danes and Olson, 2003). The negative impact of Role Clarity on firm performance in this research might suggest that Role Clarity may have an element of rigidity and is therefore detrimental to an EFBC. Finally, the contribution of Professional Networking suggests that to the extent that such activity develops human capital (Hitt et al., 2001) and enhances social capital (Sirmon and Hitt, 2003); long term firm performance is improved. Networking activity may also reflect an external orientation that supports entrepreneurship through increased knowledge, aiding opportunity recognition (Zahra et al., 2004).

Finally, investigation revealed a significant interaction effect between Family Functionality (APGAR) and Role Clarity (RCL). Results suggest that through the two-way interaction (Figures 3, 4), high Role Clarity is associated with superior firm performance in the presence of low Family Functionality; however, when Family Functionality is high, firm performance is weaker when Role Clarity is also high. So while greater Role Clarity is a common recommendation from family business research and practitioners (Dana and Smyrnios, 2010), the data suggest that high Role Clarity is not universally positive. For closely held family firms, Role Clarity may be a helpful substitute in the absence of high-functioning family owner/managers; however, when the owning family is already highly functional, high Role Clarity may stifle entrepreneurial adaptation with cumbersome or counterproductive bureaucratic structure impeding long-term performance. These findings illustrate the importance of the boundary between family and firm (Davis and Stern, 1981), where roles and rules are negotiated (Danes et al., 2008). The inclusion of the APGAR construct also addresses a need in family business research to specifically include family-related variables (Dyer, 2006).

Given the prominence of the family in the context of family owned businesses, it was anticipated that Family Functionality would play a meaningful role in firm culture; however, analysis revealed that while Family Functionality did not display a direct influence on the second-order construct—EFBC—the effect seemed to be fully absorbed by three of the independent variables; Confidence in Management, Shared Vision, and Role Clarity. Family Functionality exhibited very strong positive influence on these independent variables with highly significant path weights. While the presence and impact of family in the family firm context is foundational to the field, these findings suggest a more nuanced impact. This relationship may illustrate the manner in which family firms differ from non-family owned firms.

### Limitations

The researcher acknowledges certain limitations of this study. First, it examined only private firms that were family owned and family operated. While these companies compose a significant portion of family firms, the findings may not be generalizable to all family firms. Also, any comparison with non-family firms is beyond the scope of this research. Second, the respondents self-classified as family firms and their definition of “family firm” may not be consistent across the sample. Third, the majority of respondents were male, and data on ethnicity was not collected. The sample, therefore, may not be diverse. A fourth limitation of the study is its cross-sectional design. The theoretical focus of the SFBM is the long-term performance and sustainability of the family firm. While this study contributes to the development of the SFBM, future research using longitudinal designs may be necessary to fully understand the longer-term sustainability of family owned firms. Finally, the study examines performance perceptions over a 3-year timeframe, not actual performance over that time-frame. Respondents' performance recollections may be imperfect or biased.

### Conclusion, contribution, and future research

The results of this study have implications for theory and practice. The study identified four significant non-financial organizational traits as firm performance drivers, which addresses a need in the development of the SFBM. The performance index, composed of these traits, impacted long-term firm performance and exhibited some predictive potential. Further development of this model may provide a tool for the evaluation of private family firm performance without the need for detailed financial records which is an ongoing challenge for researchers (Mazzi, 2011). Future research using multiple respondents from each family firm may add to the understanding of findings revealed in this study. This may allow, for example, evaluation of the extent to which vision within an organization is truly shared. Also, further research is needed to fully understand the implications of the SFBM's focus on long-term sustainability rather than on long-term financial performance. Additional understanding of the dynamic between the family and business systems is needed from the perspective of long-term sustainability, such as the role of family capital (Danes et al., 2009). For practitioners who work with family firms, such findings may prove helpful in the development of family firms. For example, creation of a meaningful Shared Vision, as well as developmental and Professional Networking activity, may represent important processes for the continued success of their family firm clients.

This study represents an initial exploration and specification of some components necessary for a successful and sustainable family business. While study findings may contribute to the intermediate-term financial success of family businesses, additional work is also needed to broaden its scope to include non-financial goals of family firms, such as family harmony or satisfaction with the business. Finally, the surprising finding regarding Role Clarity, role rigidity, and family relationship dynamics merit further investigation. One possibility might be to inquire about the potential non-linear impact of Role Clarity on firm performance similar to the findings of family involvement and firm performance (Sciascia and Mazzola, 2008). In addition, the culture or ethnicity of the family may influence the flexibility or rigidity of work roles in a family business. So while this exploratory study identified some organizational traits associated with long-term family firm performance, further research is needed for a complete understanding of the long-term success of family firms.

### Conflict of interest statement

The author declares that the research was conducted in the absence of any commercial or financial relationships that could be construed as a potential conflict of interest.
