# Supplementary material for: Shared vision promotes family firm performance
Source: Front Psychol. 2015 May 19;6:646. doi: 10.3389/fpsyg.2015.00646 (PMC4436804; doi:10.3389/fpsyg.2015.00646)
Supplement: Supplementary file 1 [file DataSheet1.DOCX]

**Appendix – construct definitions and items**

| Construct | Items | Source |
| --- | --- | --- |
| Trust – Family & Organizational, using a five-point Likert scale | Benevolence:   1. My supervisor keeps my interests in mind when making decisions. 2. I would be willing to let my supervisor have complete control over my future in this company. 3. If my supervisor asked why a problem occurred, I would speak freely even if I were partly to blame. 4. I feel comfortable being creative because my supervisor understands that sometimes creative solutions do not work. 5. It is important for me to have a good way to keep an eye on my supervisor.(R) 6. Increasing my vulnerability to criticism by my supervisor would be a mistake.(R) 7. If I had my way, I would not let my supervisor have any influence over decisions that are important to me. (R)   Integrity:   1. Top management has a strong sense of justice. 2. I never have to wonder whether top management will stick to its word. 3. Top management tries hard to be fair in dealings with others. 4. Top management actions and behaviors are not very consistent. (R) 5. I like top management’s values. 6. Sound principles seem to guide top management’s behavior. | (Schoorman, Mayer, & Davis, 2007)  (Mayer & Davis, 1999) |
| Confidence in Management, using a five-point Likert scale | 1. The top management team is very capable of performing its job. 2. The top management team is known to be successful at the things it tries to do. 3. Top management has much knowledge about the work that needs to be done. 4. I feel very confident about top management’s skills. 5. Top management has specialized capabilities that can increase our performance. 6. Top management is well qualified. | (Mayer & Davis, 1999) |
| Shared Vision, using a five-point Likert scale | PEA/NEA Survey Items  Shared Vision:   1. Management emphasizes a vision for the future. 2. We often discuss possibilities for the future. 3. Our future as an organization will be better than our past. 4. I feel inspired by our vision and mission. 5. We are encouraged by management to build on our strengths. 6. Our work is focused on our mission and vision. 7. Our purpose as an organization is clear in our vision and mission. 8. Management emphasizes our current strengths.   Compassion:   1. I do not feel trusted by my colleagues. (R) 2. I feel trusted by my colleagues. 3. I care about my colleagues at work. 4. I do not trust my colleagues. (R) 5. I do not care about my colleagues at work. (R) 6. I trust my colleagues.   Overall Positive Mood:   1. This is a great place to work. 2. I enjoy working here. 3. I do not like working here. (R) 4. Working here is a joy. 5. If I had a choice, I would work somewhere else. (R) 6. Overall, it feels good to work here. | (Boyatzis, 2008) |
| Role Clarity and Role Conflict, using a five-point Likert scale | Role Clarity:   1. I feel certain about how much authority I have. 2. I have clear, planned goals and objectives for my job. 3. I know that I have divided my time properly. 4. I know what my responsibilities are. 5. I know exactly what is expected of me. 6. Explanation is clear of what has to be done.   Role Conflict:   1. I have to do things that should be done differently. 2. I receive an assignment without the manpower to complete it. 3. I have to buck a rule or policy in order to carry out an assignment. 4. I work with two or more groups who operate quite differently. 5. I receive incompatible requests from two or more people. 6. I do things that are apt to be accepted by one person and not accepted by others. 7. I receive an assignment without adequate resources and materials to execute it. | (Rizzo, House, & Lirtzman, 1970) |
| Organizational & Professional Development, using a five-point Likert scale | Commitment to Learning:   1. Managers basically agree that our organization’s ability to learn is the key to our competitive advantage. 2. The basic values of this organization include learning as key to improvement. 3. The sense around here is that employee learning is an investment, not an expense. 4. Learning in my organization is seen as a key commodity necessary to guarantee organizational survival. 5. Our culture is one that does not make employee learning a top priority. (R) 6. The collective wisdom in this enterprise is that once we quit learning, we endanger our future.   Professional Networking Behavior: “In the past I have…”   1. Given business contacts a phone call to keep in touch. 2. Attended professional seminars or workshops. 3. Attended meetings of Industry-related associations. 4. Attended meetings of civic/social groups, clubs and so forth. | (Calantone, Cavusgil, & Zhao, 2002)  (Forret & Dougherty, 2001) |
| Growth Orientation, using a five-point Likert scale | Growth Orientation:   1. The capacity of our management has kept pace with our growth. 2. We have been effective at capitalizing on opportunities. 3. We understand where our growth will come from. 4. We have the systems, procedures, and practices for significant growth.   Signs of Growth:   1. From your point of view, how important is sales volume growth as an indicator of business performance? 2. From your point of view, how important is total employment growth as an indicator of business performance? 3. From your point of view, how important is investment in capacity/technology as an indicator of business performance? | (Poza, Hanlon, & Kashida, 2004)  (Rutherford, Muse, & Oswald, 2006) |
| APGAR model of Family Functionality, using a 5 point Likert scale | Family Functionality   1. I am satisfied with the help that I receive from my family when something is troubling me. 2. I am satisfied with the way my family discusses items of common interest and shares problem solving with me. 3. I find that my family accepts my wishes to take on new activities on make changes in my lifestyle. 4. I am satisfied with the way my family expresses affection and responds to my feelings such as anger, sorrow, and love. 5. I am satisfied with the amount of time my family and I spend together. | (Smilkstein, 1978) |

**Firm level items –**

| Firm Level Characteristics  Dependent Variables, using a seven-point Likert scale | 1. Year business was founded 2. Number of generations involved in the business since business was founded 3. Number of family shareholders 4. Percentage of stock held by the family 5. Size, in percent, of largest ownership position held by an individual family member. 6. Number of family members in management in the business 7. Number of family members, who are not managers, employed in the business 8. Number of family members on the board of directors 9. Industry (NAICS categories) 10. During your fiscal year ended in 2008, indicate total company employment (FTE) from categories below   **Firm Performance vs. Competition**:   1. Relative to our Major Competitors, sales growth over the past 3 years has been VERY high. 2. Relative to our Major Competitors, our profits in the past 3 years have been VERY high. 3. Relative to our Major Competitors, our overall company growth in the past 3 years has been VERY high.   **Firm Performance vs. Historical Trend**:   1. Compared to our long term historical sales growth, our company sales growth in the past 3 years has been VERY high. 2. Compared to our long term historical profits, our company profits in the past 3 years have been VERY high. 3. Compared to our long term historical trends, our overall company growth in the past 3 years has been VERY high. | Adapted from (Habbershon, & Astrachan, 1997)  Adapted from (Rutherford, Muse, & Oswald, 2006)  Adapted from (Moreno & Casillas, 2008) |
| --- | --- | --- |
